# Time trends of cardiovascular risk management in type 1 diabetes - nationwide analyses of real-life data

**DOI:** 10.1186/s12933-022-01692-5

**Published:** 2022-11-23

**Authors:** Hanan Amadid, Kim Katrine Bjerring Clemmensen, Dorte Vistisen, Frederik Persson, Marit Eika Jørgensen

**Affiliations:** 1 Steno Diabetes Center Copenhagen, Borgmester Ib Juuls vej 83, 2730 Herlev, Danmark; 2 Steno Diabetes Center Greenland, Nuuk, Greenland

**Keywords:** Cardiovascular disease, Type 1 diabetes, Cardiovascular risk management, Cardioprotective drug use

## Abstract

**Background:**

Individuals diagnosed with and treated for type 1 diabetes (T1D) have increased risk of micro- and macrovascular disease and excess mortality. Improving cardiovascular (CV) risk factors in individuals with T1D is known to reduce diabetes- related CV complications.

**Aim:**

To examine time trends in CV risk factor levels and CV-protective treatment patterns. Additionally, examine incidence rates of diabetes-related CV complications in relation to exposure CV-protective treatment.

**Methods:**

We analysed records from 41,630 individuals with T1D, registered anytime between 1996 and 2017 in a nationwide diabetes register. We obtained CV risk factor measurements (2010–2017), CV-protective drug profiles (1996–2017) and CV complication history (1977–2017) from additional nationwide health registers.

**Results:**

From 2010 to 2017 there were decreasing levels of HbA_1c_, LDL-C, and blood pressure. Decreasing proportion of smokers, individuals with glycaemic dysregulation (HbA_1c_ ≥ 58 mmol/mol), dyslipidaemia (LDL-C > 2.6 mmol/l), and hypertension (≥ 140/85 mmHg). Yet, one fifth of the T1D population by January 1st, 2017 was severely dysregulated (HbA_1c_ > 75 mmol/mol). A slight increase in levels of BMI and urinary albumin creatinine ratio and a slight decrease in estimated glomerular filtration rate (eGFR) levels was observed. By January 1st, 2017, one fourth of the T1D population had an eGFR < 60 ml/min/1.73 m^2^. The proportion of the T1D population redeeming lipid-lowering drugs (LLDs) increased from 5% in 2000 to 30% in 2010 followed by a plateau and then a decline. The proportion of the T1D population redeeming antihypertensive drugs (AHDs) increased from 28% in 1996 to 42% in 2010 followed by a tendency to decline. Use of LLDs was associated with lower incidence of micro- and macrovascular complications, while use of AHDs had higher incidence of CVD and CKD, when compared to non-use and discontinued use, respectively.

**Conclusion:**

Improvements were seen in CV risk factor control among individuals with T1D in Denmark between 2010 and 2017. However, there is clearly a gap between current clinical guidelines and clinical practice for CV risk management in T1D. Action is needed to push further improvements in CV risk control to reduce CVD and the related excess mortality.

**Supplementary Information:**

The online version contains supplementary material available at 10.1186/s12933-022-01692-5.

## Introduction

The number of individuals with T1D is increasing worldwide. Individuals with T1D have an almost threefold higher mortality rate compared to the general population which is mainly attributed to early on development of cardiovascular disease (CVD) [1–3]. The relative risk of experiencing a coronary heart disease (CHD) event is respectively 5- and tenfold in men and women with T1D already by an age below 40 years [4]. Compared to type 2 diabetes (T2D), the incidence of CVD in T1D is similar, or even higher [2, 5, 6]. Intensified treatment of major CVD risk factors including HbA_1c_, blood pressure and low-density lipoprotein cholesterol (LDL-C) significantly lowers mortality and risk of diabetes-related CV complications among individuals with T1D, especially when multiple target levels are reached simultaneously [7–9].

Studies have documented improvements in diabetes-related CV morbidity and mortality in part due to improvements in screening, CV risk factor control for complications, and improved treatment. However, CV risk factor management is less aggressive in T1D when compared to T2D [10]. This is most likely because management of CVD risk in T1D is not examined thoroughly in randomized clinical trials and therefore relies largely on evidence for CVD risk in T2D, despite the longer duration of disease in T1D compared to T2D and evident differences in the underlying pathophysiology. Consequently, aside from glycemia management, there remains uncertainty on how aggressively CV risk factors, e.g. LDL-C, blood pressure and body mass index (BMI) should be managed. Guidelines for cardioprotective treatment are less clear in T1D compared to T2D. For this reason, CV risk, due to long diabetes duration, may be overlooked among otherwise healthy persons with T1D. Moreover, clinicians are dependent on absolute CVD risk estimates to support decisions on medical treatment for primary prevention of CVD but few CVD risk engines are available for T1D [5, 11, 12].

A first step towards evaluating barriers to better CV risk factor control is obtaining a broad assessment of CV risk management in T1D. The current status of CV risk factor management and cardioprotective drug use patterns in relation to long-term complications is largely undocumented. Time trends in CV risk-factor control may inform public health policy and guide the quality improvement work related to CV risk management in T1D. Based on nationwide register data, our study aims to examine time trends in CV risk factors and attainment of treatment goals among all individuals with T1D in Denmark in the period 2010–2017. Additionally, we describe usage patterns of cardioprotective medications and examine the association between use of cardioprotective medications and micro- and macrovascular diabetes-related complications among all individuals with T1D in Denmark in the period 1996–2017.

## Methods

This is a retrospective population-based cohort study using Danish national administrative health registers and databases. The registers are nationwide and cover all residents. Danish residents have a unique civil registration number recorded in the Danish Civil Registration System [13]. Therefore, it is possible to cross-link registers and databases at the individual level and obtain complete follow-up.

### Study population

The study population consists of individuals living in Denmark between 1996 and 2017 identified as T1D individuals based on data from the Danish health care registers and databases containing diabetes-defining information. A detailed description of the identification of individuals with T1D can be found in Carstensen et al. [2]. In short, the cohort was identified from Registry of Medicinal Products Statistics (RMPS) [14], Danish Adult Diabetes Database (DADD) [15], National Patient Register (NPR) [16], Danish National Health Service Register (NHSR) [17], and Danish Clinical Quality Assurance Database for Screening of Diabetic Retinopathy and Maculopathy (DiaBase) [18]. Individuals were classified as having diabetes with a proxy for diabetes diagnosis date being the earliest date of any of the following: (1) first occurring diagnosis of diabetes (International Classification of Diseases (ICD)-8 codes: 249 and 250; ICD-10 codes: E10 and E11; with the exclusion of gestational diabetes) in NPR (valid from 1977), (2) first occurring use of diabetes podiatry in NHSR (valid from 1990), (3) first date of purchase of any anti-diabetic medication (Anatomical Therapeutic Chemical Code (ATC) A10xxx) in RMPS (valid from 1995), (4) earliest mentioned date of diagnosis in DADD, (5) earliest date of eye examination recorded in DiaBase (valid from 2009).

Individuals were classified as having T1D if the criteria for diabetes and any of the following criteria were met; (1) purchase of insulin before age 30, (2) classified as T1D in the majority of the individual’s DADD records, (3) Not classified in DADD but with a majority of the records from NPR being classified as T1D. An individual could not be classified as having T1D if they had no recorded date of insulin purchase.

### Cardiovascular risk factors

HbA_1c_, estimated glomerular filtration rate (eGFR), urine albumin creatinine ratio (UACR), and lipid levels were sourced from the Danish National Laboratory Database (NLD) [19]. The measurements were identified by the Nomenclature for Properties and Units (NPU) codes listed in Additional file 3: Table S1. Blood pressure, BMI and smoking habits were obtained from the DADD. All cardiovascular risk factors were obtained for the period 2010–2017.

### Cardioprotective medications

Data from the RMPS was used to map cardioprotective medication usage patterns in the population for the period 1996–2017. RMPS includes all filled prescriptions since 1996 with information on ATC code and amount at the individual level. The lipid lowering drugs (LLDs) with the following ATC codes were extracted; Statins: C10AA01-07, Fibrates: C10AB01, C10AB02 and C10AB04, Bile acids: C10AC01, C10AC02 and C10AC04, Nicotinic acids: C10AD06 and C10AD52, Ezetimibe: C10AX09, PCSK9: C10AX13-14, Statin combinations: C10BA02 and C10BA05. The antihypertensive drugs (AHDs) with the following ATC codes were extracted: C02*, C03*, C07*, C08* and C09*.

Individuals were followed from 1st January 1996 or date of diabetes diagnosis until date of emigration, death or 1st January 2017. LLD-exposure was computed for the entire follow-up period using the gen.exp-function in the ‘Epi’-package in R(21). From records of drug purchase which include dates of purchase, amount purchased in number of pills and dose per time, the gen.exp-function generated LLD-exposure covariates for a particular LLD for the entire follow-up of each person. LLD exposure was assessed each 1/10 of a year time interval from start of follow-up. A grace period of 1 month was used in the definition of exposure to an LLD type, meaning that an individual was considered exposed 1 month after the end of the formally computed exposure interval. Concomitant use of different LLDs was allowed. As such, individuals could discontinue and resume therapy with LLDs several times during follow-up. The annual frequency of use for each type of LLD among the T1D population was then visualized in a bar plot.

### Cardiovascular complications

First events of diabetes-related micro- and macrovascular complications were ascertained from the National Patient Register [16]. The following complications were considered for analysis: ischaemic and haemorrhagic stroke, ischaemic heart disease, heart failure, atherosclerotic macrovascular disease, albuminuria, end-stage renal disease, lower limb amputation and moderate to severe retinopathy. A CVD composite of ischaemic and haemorrhagic stroke, ischaemic heart disease, heart failure, atherosclerotic macrovascular disease and atrial fibrillation was also defined. Events of end-stage renal disease were defined by initiation of dialysis or kidney transplantation. Measurements of albuminuria were sourced from the National Laboratory Database. NPU codes for measurements of UACR were used for ascertainment. The following thresholds were applied for classification of albuminuria; UACR < 30 mg/g as normal, UACR ≥ 30 mg/g for microalbuminuria and UACR > 300 mg/g for macroalbuminuria. Additional file 4: Table S2 supplemental data lists the specific ICD codes and Danish procedure codes used to define the disease entities.

### Emigration and death

For follow-up, death and emigration was obtained by linkage to the Central Person Register [13].

### Statistical analysis

#### Cardiovascular risk factors levels

To describe cardiovascular risk factor levels over time, we calculated the mean cardiovascular risk factor concentration for each individual per calendar year and CV risk factor type. Trends in CV risk factor levels were estimated using an additive mixed effects model containing sex, age, date of CV risk factor measurement, and duration of T1D as fixed effects and the within-individual variation as random effect. The effect of age, date of CV risk factor measurement and duration of T1D was assumed linear. Using this model, 95% prediction intervals of estimated CV risk factor values were calculated. Triglyceride and UACR values were log transformed before modelling due to skewness.

Proportions of individuals within risk factor thresholds were calculated for each year. The LDL-C thresholds were defined according to (ESC/EAS) guidelines for the management of dyslipidaemias [20]. Attainment of treatment targets are referred and compared according to the 2011/2016 version of the guidelines which were in effect at time of the study period and the most recent 2019 version. HbA_1c_ and blood pressure thresholds were defined according to the Danish Society of Endocrinology’s guidelines [21]. eGFR and UACR thresholds are according to Improving Global Outcomes (KDIGO) Guideline for Diabetes Management in Chronic Kidney Disease [22] and BMI thresholds are defined according to World Health Organization’s classification of BMI [23].

### Cardioprotective medications in relation to incidence of cardiovascular complications

During follow-up of LLD and AHD exposure, individuals could move from “never use” (the period before the first use of the drug) to “continued use” (the period(s) a person is using the drug) and at discontinuation to “discontinued use" (period(s) where the person is not using the drug, but previously has) and back to “continued use” when treatment is resumed. As such, a person can contribute with risk time in several medication exposure groups during follow-up.

Incidence rates of diabetes-related micro- and macrovascular complications by medication-exposure status (never use/continued use/ discontinued use), sex, current age, T1D duration and calendar time were estimated using Poisson regression for time-split data.

Analyses were carried out on a remote, secure server for researchers provided by Statistics Denmark with no access to the unique personal registration number. Data management was done in SAS version 9.4 and analyses were performed in R version 3.5.1.

## Results

### Study population characteristics

The study population consisted in total of 41,630 individuals with T1D in the period 1996–2017. A total of 28,401 individuals had prevalent T1D as per January 1st, 2017 of which 60% where men (Table 1).

**Table 1:** Characteristics and cardiovascular risk factor control in the T1D population during 2010–20

	2010	2011	2012	2013	2014	2015	2016	2017
N	26.180	26.426	26.680	26.959	27.294	27.658	28.090	28.401
Men	14.978	15.109	15.241	15.405	15.561	15.765	15.991	16.179
Age	45.40	45.26	45.18	45.20	45.19	45.25	45.31	45.33
Diabetes duration	14.79	15.25	15.72	16.21	16.66	17.11	17.51	17.95
Blood pressure								
N^a^	12.604	15.249	15.358	15.940	16.243	16.472	16.338	10.613
SBP ≥ 140 or DBP ≥ 85 mmHg (%)	41	37	35	35	32	32	32	34
LDL-Cholesterol								
N^a^	9.596	11.186	14.222	15.155	17.433	18.239	19.108	18.537
< 1.4 mmol/L (%)	4	5	5	6	8	8	8	9
1.4–1.8 mmol/L (%)	13	12	12	13	15	15	15	16
1.8–2.6 mmol/L (%)	44	44	43	43	42	42	42	41
2.6–3.0 mmol/L (%)	18	17	17	16	16	15	15	15
3–4.9 mmol/L (%)	21	22	22	21	19	19	19	18
≥ 4.9 mmol/L (%)	1	1	1	1	1	1	1	1
HbA_1c_								
N^a^	15.038	17.054	17.672	18.301	20.482	21.771	22.873	22.503
< 48 mmol/mol (%)	8	7	7	10	9	9	8	9
48–52 mmol/mol (%)	9	8	9	11	11	10	9	10
53–58 mmol/mol (%)	15	14	14	15	15	16	14	15
59–75 mmol/mol (%)	48	48	49	46	46	46	48	46
> 75 mmol/mol (%)	20	23	21	19	18	20	21	19
eGFR								
N^a^	2.616	5.307	8.553	14.005	15.290	16.116	19.150	18.986
≥ 90 mL/min (%)	31	32	39	48	36	35	32	32
89–60 mL/min (%)	36	35	33	32	37	37	38	37
59–30 mL/min (%)	20	19	15	12	15	16	18	18
29–15 mL/min (%)	7	8	8	5	6	5	6	7
< 15 mL/min (%)	6	6	5	3	5	6	6	6
UACR								
N^a^	11.528	13.017	12.882	13.546	14.916	15.892	16.286	15.659
Normal: < 30 mg/g (%)	80	77	75	73	72	73	72	73
Microalbuminuria: 30–300 mg/g (%)	15	17	18	21	20	20	20	19
Macroalbuminuria: > 300 mg/g (%)	5	6	6	7	8	8	8	8
Smoking								
N^a^	11.670	15.787	16.327	17.711	17.957	17.653	18.413	13.752
Daily (%)	25	26	25	22	22	21	21	21
Never (%)	54	53	56	53	54	57	57	54
Occasionally (%)	3	1	< 1	< 1	< 1	< 1	< 1	< 1
Prior (%)	19	20	20	24	24	22	22	25
BMI								
N^a^	12.244	16.670	16.929	18.159	18.480	18.113	18.825	13.988
Underweight: < 18.5 kg/m^2^ (%)	2	2	2	2	2	2	2	2
Normal weight: 18.5–24.9 kg/m^2^ (%)	50	48	48	48	48	47	46	44
Overweight: 25.0–29.9 kg/m^2^ (%)	36	37	36	36	36	37	36	37
Class I obesity: 30.0–34.9 kg/m^2^ (%)	10	11	11	11	11	11	12	13
Class II obesity: 35.0–39.9 kg/m^2^ (%)	2	2	2	3	3	3	3	3
Class III obesity: ≥ 40 kg/m^2^ (%)	1	1	1	1	1	1	1	1

^a^Annual number of prevalent individuals with one or more measurements

### Cardiovascular risk factors

**Fig. 1:**
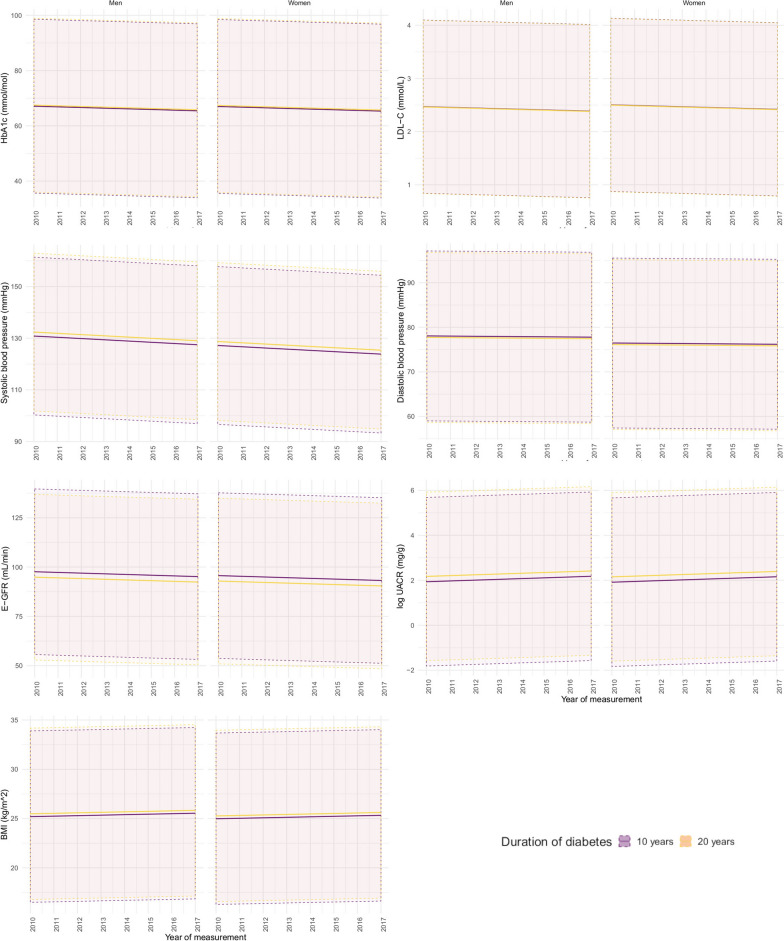
Annually estimated mean and 95% prediction interval (dotted line area) for 40 years old men and women with T1D of HbA_1c_ (**A**), LDL-C(**B**), systolic blood pressure (**C**), diastolic blood pressure (**D**), eGFR (**E**), UACR (**F**) and BMI (**G**)

From 2010–2017 decreasing levels were observed for HbA_1c_, LDL-C, and blood pressure (Fig. 1), and the proportion of smokers, individuals with glycaemic dysregulation (HbA_1c_ ≥ 58 mmol/mol), dyslipidaemia (LDL-C > 2.6 mmol/l), and hypertension (≥ 140/85 mmHg) decreased (Table 2). Yet, one fifth of the T1D population by January 1st, 2017 was severely dysregulated (HbA_1c_ > 75 mmol/mol). There has been an increase in levels of BMI and UACR and a slight decrease in eGFR levels (Fig. 1). Correspondingly, the proportion with overweight/obesity (BMI > 25 kg/m^2^), albuminuria (UACR ≥ 30 mg/g) and impaired kidney function (eGFR < 60 ml/min/1.73 m^2^) has increased (Table 2). By January 1st, 2017, one fourth of the T1D population had an eGFR < 60 ml/min/1.73 m^2^ and 54% of the population was overweight or obese.

**Table 2:** Rate ratios (RR) of diabetes-related complications according to lipid lowering drug use (A) and antihypertensive drug use (B)

	Continued vs. Never		Continued vs. Discontinued		Discontinued vs. Never	
	RR	95% CI	RR	95% CI	RR	95% CI
Cardiovascular disease	0.85	(0.73–0.98)	0.59	(0.49–0.71)	1.45	(1.21–1.73)
Cerebrovascular disease	0.91	(0.74–1.12)	0.58	(0.45–0.76)	1.56	(1.21–2.03)
Ischeamic heart disease	1.17	(0.96–1.42)	0.72	(0.56–0.92)	1.63	(1.27–2.1)
Heart failure	1.03	(0.8–1.33)	0.7	(0.51–0.98)	1.47	(1.04–2.07)
Atherosclerotic macrovascular disease	1.03	(0.8–1.34)	0.69	(0.49–0.95)	1.51	(1.08–2.12)
Atrial fibrillation	0.87	(0.67–1.13)	0.62	(0.44–0.86)	1.41	(1.01–1.99)
Chronic Kidney disease	0.9	(0.7–1.14)	0.7	(0.51–0.95)	1.29	(0.93–1.78)
Severe chronic kidney disease	1.22	(0.52–2.9)	0.72	(0.29–1.79)	1.69	(0.62–4.6)
End-stage chronic kidney disease	0.82	(0.58–1.15)	0.49	(0.33–0.73)	1.66	(1.11–2.48)
Microalbuminuria	1.04	(0.9–1.2)	0.76	(0.64–0.91)	1.36	(1.14–1.62)
Macroalbuminuria	0.97	(0.71–1.3)	0.74	(0.51–1.06)	1.31	(0.91–1.89)
Minor amputation	0.71	(0.46–1.09)	0.68	(0.39–1.2)	1.04	(0.59–1.83)
Major amputation	1.01	(0.65–1.57)	0.51	(0.31–0.85)	1.97	(1.16–3.35)
Moderate-severe retinopathy	1.01	(0.83–1.24)	0.81	(0.63–1.05)	1.25	(0.97–1.6)

	Continued vs. Never		Continued vs. Discontinued		Discontinued vs. Never	
	RR	95% CI	RR	95% CI	RR	95% CI
Cardiovascular Disease	3.08	(2.46–3.86)	0.98	(0.75–1.27)	3.15	(2.43–4.09)
Cerebrovascular disease	2.63	(1.89–3.67)	0.72	(0.5–1.03)	3.67	(2.54–5.29)
Ischeamic heart disease	2.67	(1.94–3.69)	1.3	(0.9–1.86)	2.06	(1.4–3.05)
Heart failure	8.69	(5.47–13.81)	1.16	(0.76–1.77)	7.46	(4.39–12.69)
Atherosclerotic macrovascular disease	2.88	(1.85–4.48)	1.48	(0.9–2.43)	1.94	(1.1–3.43)
Atrial fibrillation	2.89	(1.88–4.42)	1.42	(0.84–2.38)	2.03	(1.14–3.62)
Chronic Kidney disease	15.08	(9.6–23.67)	3.78	(2.23–6.4)	3.99	(2.15–7.4)
Severe chronic kidney disease	129.12	(13.99–1191.92)	4.21	(1.07–16.55)	30.68	(2.73–344.77)
End-stage chronic kidney disease	23.5	(13.63–40.5)	3.83	(2.08–7.08)	6.13	(2.97–12.66)
Microalbuminuria	1.88	(1.4–2.54)	1.08	(0.81–1.45)	1.74	(1.28–2.38)
Macroalbuminuria	13.48	(7.05–25.8)	1.74	(1.01–3.01)	7.74	(3.86–15.51)
Minor amputation	3.36	(1.56–7.24)	1.97	(0.86–4.47)	1.71	(0.66–4.41)
Major amputation	3.42	(1.74–6.75)	2.88	(1.16–7.13)	1.19	(0.43–3.28)
Moderate-severe retinopathy	1.06	(0.69–1.63)	0.62	(0.4–0.95)	1.7	(1.13–2.57)

Never use: the period before the first use of LLD; Continued use: the period(s) a person is using LLD; discontinued use: period(s) where the person is not using LLD, but previously has

### Cardioprotective drug use in relation to cardiovascular complications

**Fig. 2:**
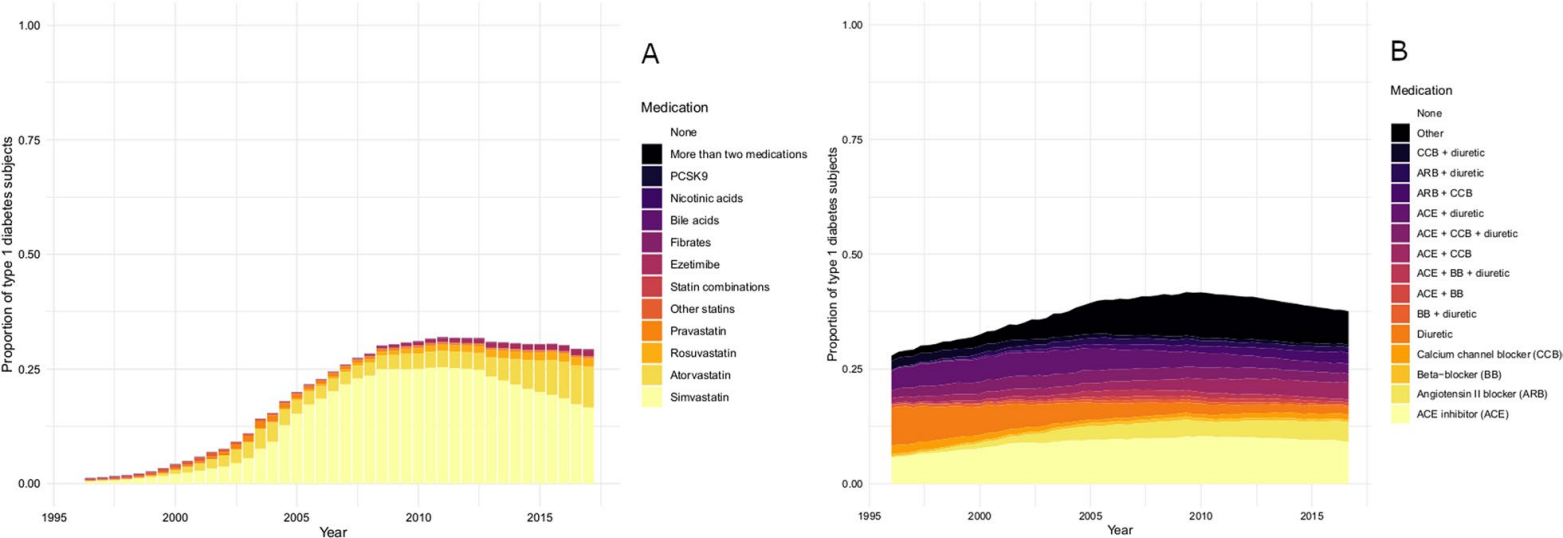
Proportion of individuals with type 1 diabetes on different lipid lowering drugs (**A**) and antihypertensive drugs (**B**) from 1996–2017. *ACE* ACE inhibitor, *ARB* Angiotensin II blocker, *CCB* Calcium channel blocker, *BB* beta–blocker, *other* all other combinations of antihypertensive drugs, that had an overall frequency of less than 2%

The proportion of the T1D population on a LLD increased from 5% in 2000 to 30% in 2010 followed by a plateau and decrease (Fig. 2A). Lipid levels were lower for LLD users compared to non-users (Additional file 1: Figure S1). 44.9% of non-users of LLDs and 16.5% of users had LDL-C levels  > 2.6 mmol/l in 2017 (Additional file 1: Figure S1). Incidence rates for CV complications were higher during discontinued use of LLD compared with individuals during never use of LLDs. Incidence rates for individuals during continued use of LLD were generally lower than for individuals during discontinued use of LLDs (Table 2A).

The proportion of the T1D population on an AHD increased from 28% in 1996 to 42% in 2010 followed by a tendency to decline (Fig. 2B). Blood pressure levels were lower among non-AHD users compared to users of AHD (Additional file 2: Figure S2). Among non-AHD users, 33.8% had blood pressure levels  ≥ 140/85 mmHg while for users of AHD, 37.5% had blood pressure levels  ≥ 140/85 mmHg (Additional file 2: Figure S2). For the vast majority of CV complications, incidence rates were higher during discontinued use of AHDs compared with individuals during never use of AHDs. In addition, continued use of AHDs had higher rates of CV complications compared with never-users. Rates of CKD for individuals with continued use of AHD were higher than discontinued and never use (Table 2B). For most complications, rates were higher among individuals during continued use AHD compared to individual during never use.

## Discussion

Improvements were seen in CV risk factor control among individuals with T1D in Denmark between 2010 and 2017. However, this study also showed that 65% of individuals with T1D had poor glycaemic control and that 34% of individuals who were not treated for hypertension had blood pressure levels above 140/85 mmHg while more than a third of individuals in AHD treatment did not reach the treatment goal. Furthermore, 45% of the T1D individuals who were not treated for dyslipidaemia had LDL-C levels above 2.6 mmol/L.

There is clearly a gap between current clinical guidelines and clinical practice. The available reporting of a significant CVD burden in individuals with T1D is to a large degree underpinned by an evidence of poor management of CV risk factors. Guidelines for the management and prevention of CV risk in individuals with diabetes have recently been updated and published by the European Society of Cardiology in collaboration with European Society for the Study of Diabetes [24]. According to the guidelines’ revised stratification of CV risk, most individuals with T1D but without established CVD can be considered at high or very high CV risk [24]. The clinical implications of the new CV risk categories imply that not only more aggressive but also more ambitious targets for the multifactorial approach have been set for the management of LDL-C (changing from < 1.8 mmol/L (70 mg/dL) to < 1.4 mmol/L (55 mg/dL), blood pressure control (changing from SBP < 140 mmHg to < 130 mmHg) and the use of antiplatelet agents, also among the younger individuals with T1D.

### Achieving target HbA_1c_

During the last decades, improved glycaemic control have been reported corroborating our findings. In a nation-wide Scottish study, median HbA_1c_ decreased from 72 mmol/mol in 2004 to 68 mmol/mol in 2016 in adults with T1D [25]. Similar to international diabetes associations, the Danish Endocrinology Association recommends a target HbA_1c_ of 48–58 mmol/mol (6.5–7.5%) for most individuals with T1D [21]. However, as we and others report, despite improvements in HbA_1c_, the majority of the T1D population is still far from attaining HbA_1c_ target levels (65% had a HbA_1c_ > 58 mmol/mol (7.5%)).

### Achieving target blood pressure

Hypertension is strongly associated with an increased risk of microvascular complications, such as retinopathy, nephropathy, as well as CV diseases including ischaemic heart disease, stroke and heart failure [26]. We found that the proportion of T1D individuals reaching the treatment goal improved during the study period and that antihypertensive drug use was associated with lower risk of CVD, cerebrovascular disease and chronic kidney disease. However, it should be pointed out that the treatment goal was not achieved in more than one-third of the population in 2017. Of note, we found that persons in continuous AHD treatment had higher CV and CKD incidence as compared to never-users, probably reflecting the fact that AHD treatment is prescribed not only for hypertension, but also for heart failure and CKD, explaining the association with these outcomes. The overall AHD use in the Danish T1D population showed an unexpected decline in the year 2012 and onward. A recent analysis of the National Health and Nutrition Examination Survey data showed that the prevalence of controlled blood pressure and users of antihypertensive drugs in the US have decreased in the general population from 2013 to 2018 and parallel trends were found in the diabetes population [27, 28].

### Achieving target lipid levels

Contemporary data indicate that clinicians may be undertreating dyslipidaemia among individuals with T1D(30). LLD treatment is prescribed far less to those with T1D specifically to those who are considered young (< 40 years). However, there is evidence that primary CVD prevention among individuals with T1D, especially younger individuals, may be more beneficial to these individuals if a more aggressive treatment was initiated earlier in the course of T1D. This is an important clinical point because currently LLDs are less commonly used in individuals with T1D who are  < 40 years of age. Our follow-up for micro- and macrovascular complications and exposure to LLD use showed lower rates of various CV complications during LLD use, ischaemic heart disease, heart failure, cerebrovascular disease and chronic kidney disease (Table 2).

The findings of our study are consistent with previous studies that have reported undertreatment of hypertension and dyslipidaemia especially among individuals with T1D. Even though the causes are multifaceted, there are main factors at the clinician-, patient- and system-level influencing the delay in initiation, continuation or intensification of the drug treatments which exposes the individuals to unnecessary prolonged periods of high CV risk. One factor is that consolidated evidence from randomized clinical trials and observational studies is needed for translation into a clear set of guidelines on how to manage CV risk, which clearly distinguish recommendations for T2D from those for T1D. Another major barrier is a limited awareness among clinicians of clinical inertia, resulting in lack of evaluation of the quality of CV risk management and patient’s adherence to treatments. Finally, perceptions among individuals about adverse effect due to LLDs and AHDs result in non-adherence to these therapies. We have prior found in a population with T2D that poor initiation rather than poor implementation by the clinicians or discontinuation was the main contributor to LLD and AHD nonadherence [29]. Whether this is the case in individuals with T1D needs to be examined.

### Overweight

Individuals with T1D have traditionally, been considered to have normal BMI. Studies have reported that the prevalence of overweight or obesity is increasing in T1D, in some studies rates were even higher than in the general population. Among 641 men with T1D in the Epidemiology of Diabetes Interventions and Complications study, more than 78% were overweight and obese [30]. An Australian study with 501 adults with T1D found that more than 50% were overweight or obese, and approximately 15% were obese [31]. We found similar proportions of overweight/obesity in the Danish T1D population as per 2017. Moreover, the trend was found to be increasing since 2010. Obesity is associated with various metabolic abnormalities which challenge not only the benefits of good glycaemic control which in turn are likely to modify CVD risk in this population. Weight management in T1D is complex and recommendations need to account for the competing outcomes of optimal glycemic control and weight management.

### Smoking status

Smoking accelerate vascular damage in T1D. In Denmark, as in many European countries, the prevalence of individuals who smoke had decreased significantly during the study period, and this trend has occurred also among Danish individuals with T1D. In contrast, studies from other countries, e.g. the US show that trends smoking cessation guided by structured advise is urged in all individuals with diabetes.

### Implications

Our findings raise several issues and provide important basis for actions toward improving the CV health of individuals with T1D. Implementing the new guidelines will require an additional effort to achieve the treatment targets. This will include enforcement of the guidelines, more consequent in screening for CV risk factors as well as early and effective intervention. Currently, there is widespread global implementation of hybrid closed-loop systems that are efficient when it comes to achieving better glucose control, which may well transform CV risk for recently diagnosed persons with T1D. This could limit the need for strict risk factor control, but even though updated technologies and new glucose-lowering drugs are gaining ground as treatment modalities in T1D, we believe that the potential of the conventional first-line therapies for CVD prevention should be exploited fully as a first step. It may also be that the currently suggested risk factor targets are too strict, as they are based on observational studies and extrapolated evidence from T2D. Therefore, there is a clear need for randomized, controlled trials in T1D to evaluate optimal treatment goals and effectiveness of cardioprotective medications in T1D individuals at CV risk.

### Strengths and limitations

The strength of our study lies in the complete nationwide population-based register data with repeated CV risk measures and two decades of cardioprotective drug use information and diabetes-related complication history which allowed us to estimate long-term CV risk factor trends and relate cardioprotective drug use patterns to both CV risk factor levels and diabetes-related micro- and macrovascular complication rates. Because of the administrative nature of the Danish registries there is no loss to follow up and data validity is high. We could reliably distinguish between different types of diabetes allowing us to focus on T1D. Limitations were that laboratory measurements have only been available since 2010. Also, since data from the RMPS contain information on redeemed prescriptions only, we are not able to determine whether the declining trends in usage of LLDs and AHDS is due to individuals not redeeming their prescription or physicians not adequately prescribing LLDs and AHDs.

## Conclusion

Despite the encouraging improvement in levels of most CV risk factors during the last decade, only a minority of Danish individuals with T1D achieve national and international treatment goals for HbA_1c_, blood pressure and LDL-C. Use of LLDs and AHDs declined after 2012. This demonstrates the need for a greater awareness and more aggressive attitude among physicians in delivering and sustaining CV risk management in T1D individuals to ultimately prevent both the morbidity and years of life lost from CVD among individuals with T1D.

## Supplementary Information

**Additional file 1: Figure S1. **Proportions within LDL-C thresholds among the type 1 diabetes population by lipidlowering drug use.

**Additional file 2: Figure S2. **Proportions within blood pressure thresholds among the type 1 diabetes population by anithypertensive drug use.

**Additional file 3: Table S1. **Risk factors and corresponding Nomenclature for Properties and Units codes.

**Additional file 4: Table S2. **International Classification of Diseases 10th revision (ICD-10) codes and Danish procedure codes used to define micro- and macrovascular complications.

## Data Availability

The data that support the findings of this study are available from the Danish Data Protection Agency and Danish Health Data Agency (j-No: SDC-2017-026 and FSEID-00003715) but restrictions apply to the availability of these data, which were used under license for the current study, and so are not publicly available.

## Abbreviations

ATC: Anatomical therapeutic chemical code
BMI: Body mass index
CKD: Chronic kidney disease
CPR: Civil personal register
DADD: Danish adult diabetes database
DiaBase: Database for screening of diabetic retinopathy and maculopathy
HbA1c: Haemoglobin A1c
ICD-8: International classification of diseases, 8th revision
ICD-10: International classification of diseases, 10th revision
eGFR: Estimated glomerular filtration rate
GFR: Glomerular filtration rate
NHSR: National Health Service Register
NLD: National laboratory database
NPR: National patient register
NPU: Nomenclature for properties and units
RMPS: Register of medicines products statistics
T1D: Type 1 diabetes
T2D: Type 2 diabetes
