# Supplementary figures and images for: Time trends of cardiovascular risk management in type 1 diabetes - nationwide analyses of real-life data
Source: Cardiovasc Diabetol. 2022 Nov 23;21:255. doi: 10.1186/s12933-022-01692-5 (PMC9685843; doi:10.1186/s12933-022-01692-5)

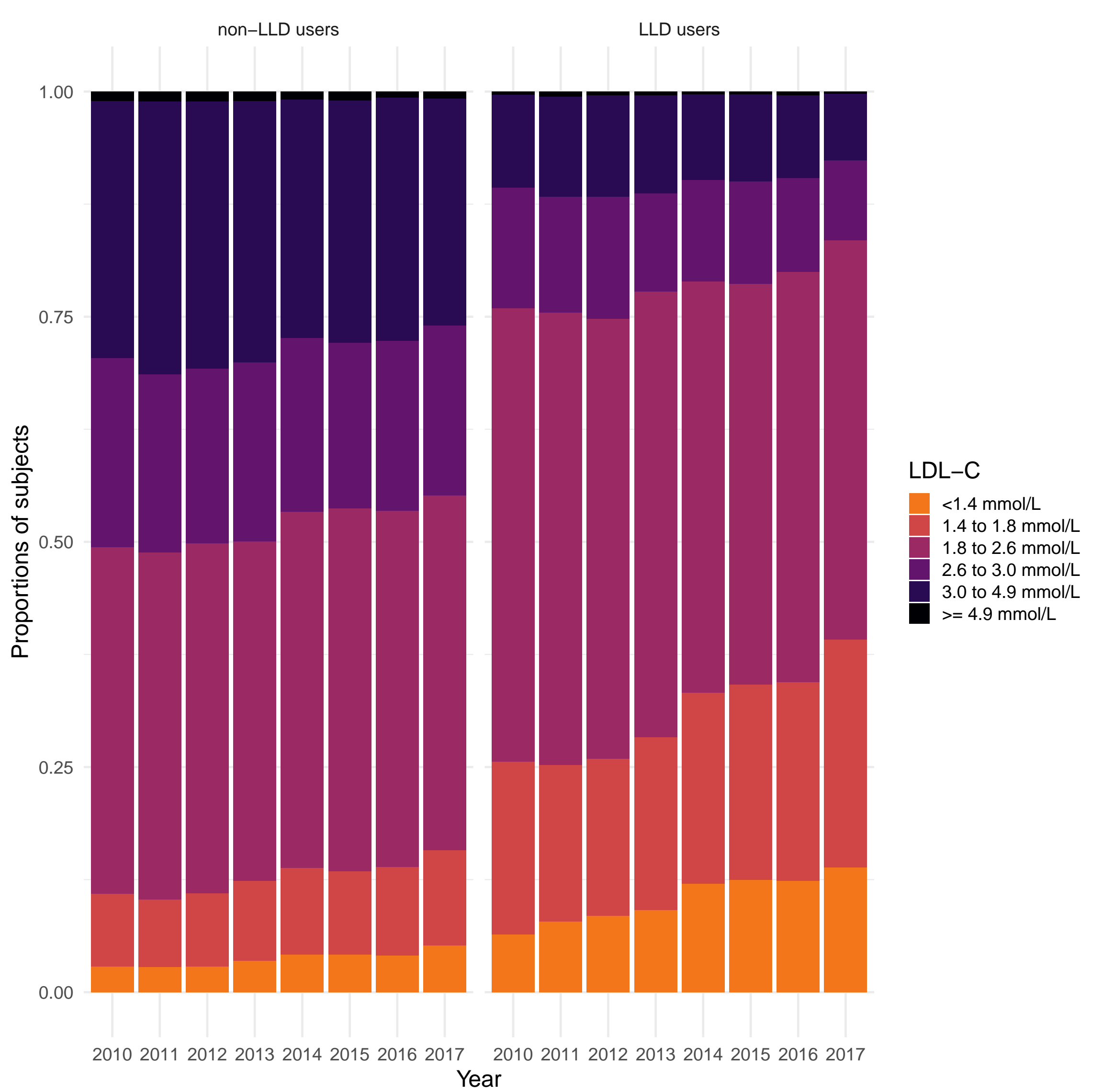

Supplement: Supplementary file 1 — Additional file 1: Figure S1. Proportions within LDL-C thresholds among the type 1 diabetes population by lipidlowering drug use. [file 12933_2022_1692_MOESM1_ESM.pdf]

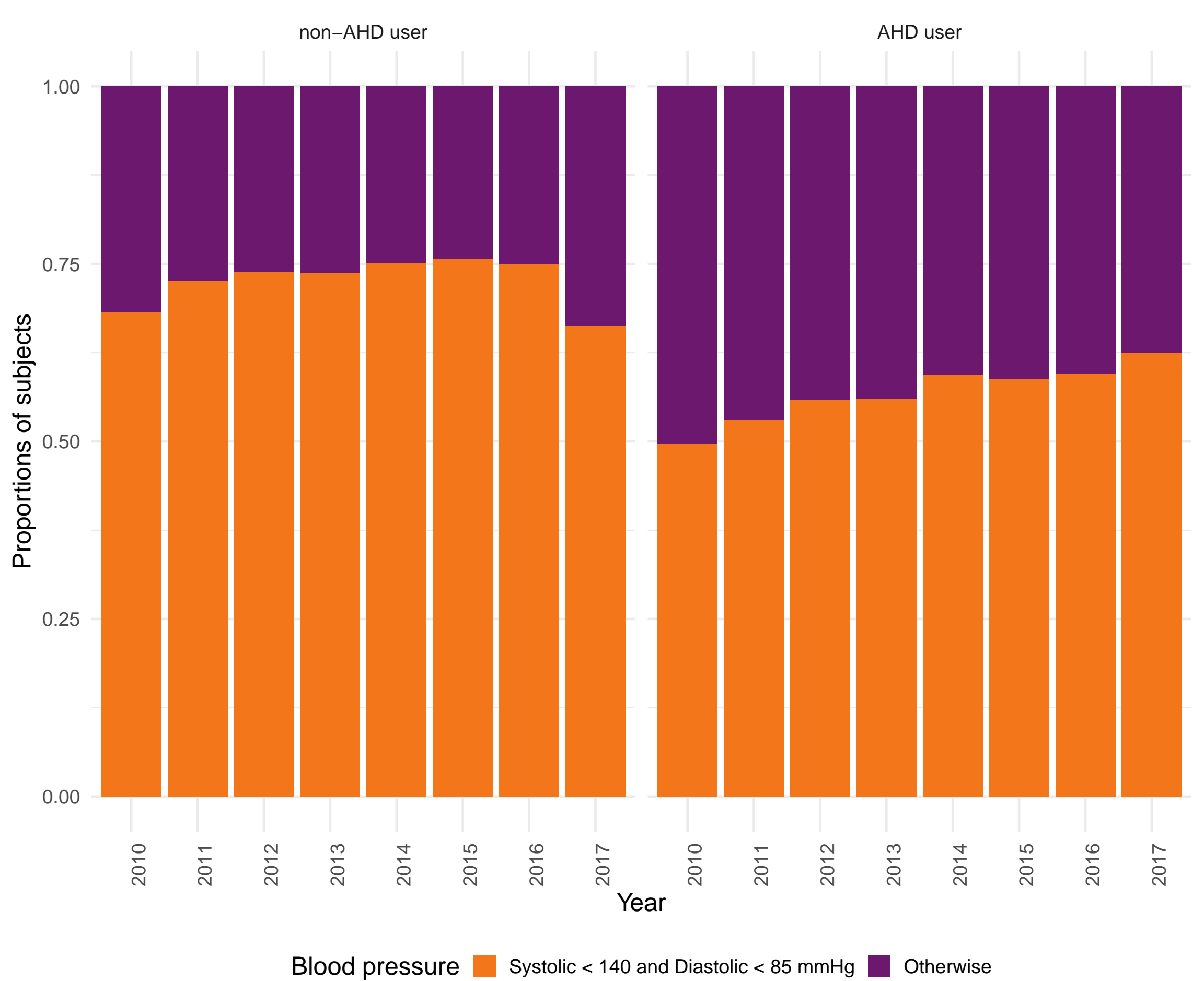

Supplement: Supplementary file 2 — Additional file 2: Figure S2. Proportions within blood pressure thresholds among the type 1 diabetes population by anithypertensive drug use. [file 12933_2022_1692_MOESM2_ESM.pdf]
