# Supplementary material for: Time trends of cardiovascular risk management in type 1 diabetes - nationwide analyses of real-life data
Source: Cardiovasc Diabetol. 2022 Nov 23;21:255. doi: 10.1186/s12933-022-01692-5 (PMC9685843; doi:10.1186/s12933-022-01692-5)
Supplement: Supplementary file 3 — Additional file 3: Table S1. Risk factors and corresponding Nomenclature for Properties and Units codes. [file 12933_2022_1692_MOESM3_ESM.docx]

| Risk factor | Nomenclature for Properties and Units codes |
| --- | --- |
| HbA1c | NPU27300  DNK35249  NPU29296  NPU03835  NPU02307 |
| eGFR/GFR | DNK35131  NPU28811  DNK35301  DNK35302  DNK35303  DNK35304  NPU19597  NPU28271  NPU10295 |
| LDL-C | NPU10171  NPU01568  DNK35308 |
| UACR | NPU28842  DNK05289  NPU19661  NPU03918 |

**Supplemental table S1. Risk factors and corresponding Nomenclature for Properties and Units** **codes**
