# Supplementary material for: Time trends of cardiovascular risk management in type 1 diabetes - nationwide analyses of real-life data
Source: Cardiovasc Diabetol. 2022 Nov 23;21:255. doi: 10.1186/s12933-022-01692-5 (PMC9685843; doi:10.1186/s12933-022-01692-5)
Supplement: Supplementary file 4 — Additional file 4: Table S2. International Classification of Diseases 10th revision (ICD-10) codes and Danish procedure codes used to define micro- and macrovascular complications. [file 12933_2022_1692_MOESM4_ESM.docx]

**Table S2. International Classification of Diseases 10th revision (ICD-10) codes and Danish procedure codes used to define micro- and macrovascular complications.**

|  | **ICD-10 codes** | **Procedure codes** | **Laboratory measure values** |
| --- | --- | --- | --- |
| **Ischemic heart disease** | DI20*  DI21*  DI23*  DI24*  DI25* | KFNA*  KFNB*  KFNC*  KFND*  KFNE*  KFNF*  KFNG* (-KNFG 20+22) |  |
| **Stroke** | DI60*  DI61*  DI62*  DI63*  DI64*  DI65*  DI66*  DI67*  DI68*  DI69*  DG45* |  |  |
| **Macrovascular atherosclerotic disease** | DI70* DI71* DI739A+C |  |  |
| **Heart failure** | DI50*  DI110 |  |  |
| **Atrial fibrillation/flutter** | DI48* | "KFPD00 |  |
| **Chronic Kidney Disease** 1.       Moderate CKD  2.       Severe CKD  3.       End Stage CKD (incl. dialysis and kidney transplantation) | DN183  DN189  DN184  DN185 | "BJFD*  BJFZ*  KPBL*  KJAK10  KJAK11  KJAK13  KJAK14" | “**GFR 30-59 mL/min**  *second successive recorded GFR with >2 month apart between measures "  "**GFR 15-29 mL/min**  *second successive recorded GFR with >2 month apart between measures "  "**GFR < 15 mL/min**  *second successive recorded GFR with >2 month apart between measures " |
| **Albuminuria**  Microalbuminuria      Macroalbuminuria |  |  | **UACR 30-300 mg/g**  *second successive recorded UACR    **UACR >300mg/g**  *second successive recorded UACR |
| **Retinopathy** | "DH33*  DH35.2  DH360J  DH360K  DH368D1  DH368D2  DH420  DH43* | KCKB*  KCKC*  KCKD*  KCKE*  KCKW*  BCDE  BCHY8A |  |
| **Amputations**  **Major:**    **Minor:** | DZ897  DZ896  DZ895  DZ894 | KNFQ09  KNFQ19  KNFQ99  KNGQ09  KNGQ19  KNGQ99  KNHQ0*  KNHQ1*  KNHQ99***** |  |
